# Are ADHD medications under or over prescribed worldwide? Protocol for a systematic review and meta-analysis

**DOI:** 10.1097/MD.0000000000010923

**Published:** 2018-06-15

**Authors:** Carlos Renato Moreira-Maia, Rafael Massuti, Luca Tessari, Fausto Campani, Glaucia Chiyoko Akutagava-Martins, Samuele Cortese, Luis Augusto Rohde

**Affiliations:** a ADHD Outpatient Program (PRODAH), Child and Adolescent Psychiatry Division, Hospital de Clínicas de Porto Alegre, Federal University of Rio Grande do Sul, Porto Alegre, Brazil; b Department of Psychology, Centre for Innovation in Mental Health, University of Southampton; Clinical and Experimental Sciences (CNS and Psychiatry), University of Southampton; c College of Medicine, Universidade Federal de Mato Grosso, Cuiabá, Brazil.; d Solent NHS Trust, Southampton, UK; e New York University Child Study Center, New York, NY; f Division of Psychiatry and Applied Psychology, School of Medicine, University of Nottingham, Nottingham, UK; g National Institute of Developmental Psychiatry for Children and Adolescents, Porto Alegre, Brazil.

**Keywords:** ADHD, attention-deficit/hyperactivity disorder, meta-analysis, overtreatment, protocol, stimulants, Systematic review

## Abstract

**Introduction:**

Attention-Deficit/Hyperactivity Disorder (ADHD) is a common neurodevelopmental disorder, characterized by age inappropriate and impairing levels of inattention and/or hyperactivity/impulsivity. Pharmacotherapy is an important part of the ADHD multimodal treatment. The extent to which ADHD is pharmacologically over or under treated worldwide is controversial. We aimed to estimate the pooled worldwide rate of ADHD pharmacological treatment in individuals with and without the disorder.

**Method and Analysis:**

We will include published or unpublished studies reporting the rates of ADHD pharmacological treatment in participants with and without ADHD of any age group. Population-based, cohort, or follow-up studies, as well as data from insurance health system and third-party reimbursements will be eligible. Searches will be performed in a large number of electronic databases, including Medline, Embase, CINAHL, Cochrane, PsycINFO, Web of Science, and Scopus. The primary outcome will be the prevalence of ADHD pharmacological treatment in individuals with ADHD and without ADHD. Two independent reviewers will perform the screening, and data extraction process. Study quality/bias will be assessed with the Newcastle–Ottawa scale by 2 independent reviewers. To test the robustness of the findings, we will perform a series of sensitivity and meta-regression analysis. Analyses will be performed with R and STATA software.

**Ethics and Dissemination:**

No IRB approval will be necessary. The results of this systematic review and meta-analysis will be presented at international conferences and published in peer-reviewed journals.

**Registration and Status:**

PROSPERO 2018 CRD42018085233.

## Abstract 2

Supplemental Digital Content is available in the text

## 1 Introduction

Attention-Deficit/Hyperactivity Disorder (ADHD), one of the most common neurodevelopmental disorders, is characterized, as per the Diagnostic and Statistical Manual of Mental Disorders, fifth edition,^[1]^ by an age inappropriate and impairing pattern of inattention and/or hyperactivity/impulsivity. The ADHD worldwide prevalence is estimated at around 5% and 2.5% in children/adolescents and adults, respectively.^[2–4]^ Hyperkinetic disorder (HKD) as per the International Classification of Diseases, tenth edition^[5]^ is a more restrictive syndrome, requiring symptoms and impairment in both the inattention and hyperactivity-impulsivity domains, thus roughly equivalent to DSM-5 ADHD combined presentation. Its prevalence is estimated at around 2%.^[6]^

Pharmacotherapy is part of the multimodal therapeutic strategy for ADHD and it is recommended as the first-line option in the most commonly used guidelines/practice parameters,^[7–9]^ at least for severe cases,^[10]^ or as a treatment strategy for patients who have not responded to nonpharmacological interventions.^[10,11]^ Medications for ADHD include psychostimulant (e.g., methylphenidate and amphetamines) and nonpsychostimulant drugs (e.g., atomoxetine or guanfacine).

Currently, there is a controversy as to whether ADHD is under or over diagnosed^[12]^ and, related to this, if it is under or over treated with medications. Indeed, evidence from several countries shows that there has been an increase, over the last decades, in the prescription rate of ADHD medications.^[13–16]^ However, to what extent the increase in prescription rates can be considered an “overprescribing phenomenon” remains to be elucidated.^[12]^

Additionally, regardless of the possible increased prescription rate, it is not clear to which extent all individuals with ADHD, who would benefit from a pharmacological treatment of ADHD, indeed receive prescriptions of ADHD medications. Considering that some guidelines such as the National Institute for Health and Clinical Excellence (NICE) only recommend a pharmacological treatment for the most severe form of ADHD (i.e., HKD),^[17]^ prescription rates of ADHD medications would be expected at minimum around 2%. On the one hand, prescription rates higher than the worldwide prevalence of a more flexible ADHD definition, i.e. the DSM definition (5.9%–7.1%)^[18]^ would necessarily indicate an overtreatment, with possible unnecessary side effects. On the other hand, prescription rates lower than the prevalence of ADHD according to the more conservative ICD (International Classification of Diseases) definition (2%) would suggest that a portion of patients with ADHD are not benefitting from potentially useful medications. Indeed, ADHD may result in several serious consequences if left untreated. Children and adolescents might present a 1.53 risk of being unintentionally uninjured than controls.^[19]^ Similarly, the risk for car accidents for ADHD licensed drivers was found to be 1.36 times higher than for non-ADHD.^[20]^ As a consequence, the disorder is associated with a mortality rate of 5.85 per 10,000 person-years, mostly caused by accidents, whereas the rate in nonaffected subjects is around 2.21 per 10,000 person-years.^[21]^ Evidences show that the pharmacological treatment for ADHD can positively impact patients’ lives. Regular treatment can lower the risk of substance use among hyperactive/impulsive and combined subtypes,^[22]^ as well as the risk of car accidents,^[23,24]^ unintentional^[19]^ injuries, emergency department visits,^[25]^ depression,^[26]^ and suicide.^[27]^ The medication can also benefit school work, classroom behavior,^[28]^ and reduce rates of criminality.^[29]^

Therefore, addressing the question: “is ADHD under or over treated worldwide?” is a major public health need. To our knowledge, no systematic review with meta-analysis has been conducted to estimate the pooled rates of ADHD medications prescription worldwide. We aimed to fill this important gap in the literature. Here, we present the protocol of this systematic review/meta-analysis.

## 2 Objectives

The overarching aim of this systematic review and meta-analysis is to estimate the worldwide prevalence of ADHD pharmacological treatment. We aimed to address the following primary questions: What is the prevalence of individuals with ADHD receiving and not receiving pharmacological treatment? What is the prevalence of individuals without ADHD receiving and not receiving ADHD treatment? Is there any significant difference in the rates of ADHD pharmacological treatment worldwide? In addition, we also aimed to address the following secondary questions: Are ADHD patients receiving doses of medications as recommended in international guidelines? What is the prevalence of individuals treated with ADHD medications for 3 or more weeks? We choose this time length based on the minimum time of response and efficacy^[30]^ to treatment in clinical trials.

## 3 Method

The proposed systematic review and meta-analysis will be developed in accordance with the recommendations of the Preferred Reporting Items for Systematic Reviews and Meta-Analyses (PRISMA),^[31]^ and the Meta-Analysis of Observational Studies in Epidemiology (MOOSE).^[32]^ This protocol has been registered in the International Prospective Register of Systematic Reviews (PROSPERO), ID: CRD42018085233.^[33]^

### 3.1 Eligibility criteria

#### 3.1.1 Types of studies

We will include published and unpublished population-based, cohort, or follow-up studies (independent of the length of treatment). Data from insurance health system and third-party reimbursements will also be eligible. For longitudinal studies, we will include data from the first wave, when available, or at the earliest time point. Randomized and nonrandomized clinical trials and studies only reporting prevalence or prescription rate of ADHD treatments without an ADHD diagnosis will be excluded.

#### 3.1.2 Population

Individuals from all age groups (children [< 12 years], adolescents [≥12 year and < 18 years], and adults [≥18]) with a primary diagnosis of ADHD, ADHD-NOS (not otherwise specified), and ADHD-U (unspecified) or HKD determined as follows: categorical diagnosis as per DSM-II,^[34]^ DSM-III,^[35]^ III-R,^[36]^ IV,^[37]^ IV-TR^[38]^ or 5,^[1]^ (any subtype) or ICD-9,^[39]^ and 10,^[5]^ or according to diagnostic instruments (Supplemental Digital Content 1); the presence of symptoms above a prespecified threshold on a validated ADHD questionnaire (Supplemental Digital Content 2); diagnosis of ADHD reported by participants and/or their caregiver (e.g., positive answer to questions such as “does any doctor has diagnosed you [or your familiar] with ADHD?”); diagnosis recorded in medical files or registers from health care agencies.

Studies including *some* ADHD participants with psychiatric comorbidities will be retained. However, we will exclude studies where *all* participants have ADHD plus one or more psychiatric comorbidities, since this may impact the rate of ADHD medications prescriptions (e.g., patients with ADHD plus autism spectrum disorder generally tend to be prescribed ADHD medications less frequently, due to concerns around side effects). Similarly, studies that included some ADHD patients with IQ (intelligence quotient) lower than 70 or with neurological comorbidities will be accepted. If feasible, a sensitivity analysis excluding studies in which some participants have IQ < 70 and/or psychiatric/neurological comorbidities will be performed. Studies reporting ADHD medication misuse and abuse will be selected only if participants were accessed for ADHD diagnosis as described above.

Controls will be considered as those individuals without any neuropsychiatric diagnosis.

#### 3.1.3 Interventions

We will consider studies on first and second line ADHD medications, according to the most commonly used international guidelines: American Academy of Child and Adolescent Psychiatry,^[7]^ American Academy of Pediatrics,^[11]^ Canadian Attention Deficit Hyperactivity Disorder Resource Alliance,^[9]^ The European Network Adult ADHD,^[8]^ European Network for Hyperkinetic Disorders,^[10]^ and the National Institute for Health and Care Excellence,^[40]^that is, psychostimulants (methylphenidate [immediate, modified, or extended release], dexmethylphenidate, amphetamines [including dexamphetamine, mixed amphetamine salts, and lisdexamfetamine]) and, among nonpsychostimulants, atomoxetine. In the case of studies not reporting the number and/or percentage of ADHD individuals with/without treatment, we will contact study authors via e-mail to gather these data.

We will exclude studies where the method to diagnose ADHD is not clear, even after contacting/attempting to contact the authors. We will also exclude studies using the pharmacological treatment of ADHD as a proxy for diagnosis since this will create a clear, misleading tautological approach to answer our research questions (i.e., studies based on the number of prescriptions claims, dispensing rates; or studies based on prescription habits of general practitioners and specialists).

#### 3.1.4 Outcomes

The primary outcome will be the worldwide prevalence of treatment with psychostimulants or atomoxetine in subjects with or without ADHD, ADHD-NOS (not otherwise specified) or ADHD-U (unspecified). For “treatment,” we will consider 2 approaches: any reported dose of the ADHD medications, and only the use of ADHD medications for 3 weeks or more.

The secondary outcomes are prevalence of individuals receiving the recommended doses according to the international guidelines and, if feasible, the prevalence of ADHD medication use, considering treatment for 3 weeks or more.

### 3.2 Information sources and search method

Searches will be performed by a health Librarian at the University of Southampton, UK, who will work along with the authors to develop the search strategy. The search will be performed on the following databases using the same keywords and amending the Subject Heading as appropriate: Medline, Embase, CINAHL, PsychINFO, Web of Science, and Scopus. (The detailed individual search strategies and syntax for each database are reported in Supplemental Digital Content 3). The Pubmed syntax used will be: (minimal brain disorder OR minimal brain dysfunction OR overactive child syndrome) OR adhd OR ADHD OR addh OR ADD OR attention deficit (disorder∗ OR syndrome∗) (hyperactiv∗ OR hyperkinetic∗) OR (MH “Attention Deficit Disorder with Hyperactivity) AND (Amphetamine∗ OR Amfetamine∗ OR Dextroamphetamine∗ OR Dexamphetamine∗ OR ”Mixed amphetamine salts“ OR Lisdexamfetamine∗ OR Methylphenidate OR Atomoxetine OR Clonidine OR guanfacine OR stimulant∗ OR psychostimulant∗ OR Elvanse OR Venvanse OR Adderall OR Dexedrine OR Detrostat OR Vyvanse OR ProCentra OR Dyanavel OR Evekeo OR Zenzedi OR Desoxyn OR Metadate OR Concerta OR Daytrana OR Ritalin OR Methylin OR Quillivant OR Focalin OR Biphentin OR Phenida OR Ritalina OR Hynidate OR Addwize OR Inspiral OR Attenade OR Medikinet OR Equasym OR Penid OR Tranquilyn OR Rubifen OR Aptensio OR Strattera OR Tomoxetin OR Attentrol OR Axepta OR Atoken OR Attentin OR Kapvay OR Intuniv) OR (pharmacological treatment∗ OR drug∗ treatment∗ OR pharmacotherapy OR psychotropic drug∗ OR medicat∗ OR [MH Psychotropic Drugs]) AND (cohort n (study or studies)) OR ”cohort analy∗“ OR (follow up n (study or studies)) OR (observational n (study or studies)) OR longitudinal OR retrospective OR (epidemiological n [study OR studies]) OR (”cross section“ n [study OR studies]) OR ”cross sectional“ OR ”follow up“ OR [MH Health Care Surveys]) AND (incidence OR prevalence OR occur∗ OR frequenc∗ OR proportion∗ OR rate∗ OR number∗ OR percent∗ OR episode∗ OR epidemiolo∗ OR distribut∗ OR demograph∗ OR survey∗ OR trend∗) AND ([MSH ”Epidemiologic Methods“] OR [MSH ”Incidence“] OR [MSH ”Prevalence“] OR [MSH ”Demography“] OR [MSH Epidemiology])).

#### 3.2.1 Additional database searching

Using only keywords, the following databases will be searched as part of the University of Southampton EBSCO Discovery Portal (for more information on these sources: http://library.soton.ac.uk/resources): Business Source Premier; Research Starters; SciELO; AMED; Cochrane Database of Systematic Reviews; PsyArticles; Teacher Reference Centre; Eprints Soton; British Library Ethos; Science Citation Index; Social Sciences Citation Index; Science Direct; OAster; ERIC; SocIndex with Full text; Sports Discuss; RePec; Green File; PsycCRITIQUES. We will also search 25 additional sources (List of websites are in Supplemental Digital Content 4). For this search, the following keywords will be used: “ADHD,” “prevalence,” “survey,” “trend,” “pharmacological,” “medication,” “epidemiology,” “attention deficit,” where only simple searches could be undertaken. We will not use any language or date restrictions.

#### 3.2.2 Other sources

We will hand-search the reference list of papers selected for full text reading. Similarly, we will perform a manual search in the reference list of systematic reviews and meta-analysis on ADHD treatment retrieved in the electronic search. In addition, we will contact each first study author of retained studies and experts in the field and we will search the ProQuest database of thesis and dissertations in order to reach information of ongoing and/or unpublished studies.

### 3.3 Identification and selection of studies

#### 3.3.1 Data management

After the search process, all abstracts will be uploaded to Covidence software (https://www.covidence.org/). Two independent reviewers (LT and RM) will conduct the screening of titles and abstracts. Selected titles will have the full text included for a careful reading by 3 independent reviewers (GCAM, LT, and RM), thus ensuring that the study really fulfil the inclusion criteria. In this process, a third experienced researcher (CRMM) will evaluate and resolve any disagreement in the process whenever is necessary.

#### 3.3.2 Data collection process

Each study will be stored in Google Drive, which will contain one or more pdf files (depending on the number of publications for each study) and a data extraction sheet. Independent reviewers working in pairs (LT and RM) will collect and compare the data extraction sheets. Discrepancies in the process will be resolved between the 2 reviewers. In case of no consensus, 3 senior researchers (CRMM, LAR, and SC) will resolve the disagreement in the process. At this phase, authors from the selected studies will be contacted for additional information or clarification whenever necessary. After 3 failed attempts to contact the authors via e-mail, the study will be discarded.

Studies reported in more than one publication will be identified as one entrance, and we will provide an appropriate description in the text. In these cases, the preference will follow this order: the first publication of the study, the largest sample size, and the most complete data.

Reviewers will collect a pilot sample of 10 studies in order to test the integrity of the process of collecting and analysing the data.

To assure the integrity of the data collection process, 2 independent reviewers (CRMM and GCAM) will transfer the data from extraction sheets to Excel tables, which will pass by a double-conference process. Discrepancies in this process will be resolved between the 2 reviewers.

### 3.4 Data extraction

The following information will be extracted:

1. *Study*: Author, year of publication, country, continent, year of data collection, name, if any, given to the sample (e.g., 2009–2010 National Survey of Children with Special Care Needs, NS-CSHCN), and study design.
2. *Patient*: age range, age mean, gender (n and %), method of diagnosis (physician/reported by caregivers), diagnostic criteria (DSM/ICD), comorbidities (% and type), co-medication, name of diagnostic questionnaire and rating scale (if described in the paper), socioeconomic status, level of care (primary, secondary, or tertiary).
3. *Intervention*: medication class and formulation, dose range, mean dose (reported as mg/kg/day, or mg/day), and length of treatment (reported in days, weeks, or years).
4. *Primary outcomes*: the total number of participants with ADHD, ADHD-NOS, ADHD-U and non-ADHD diagnosis receiving and not receiving ADHD pharmacological treatment, number of participants without any ADHD diagnosis receiving and not receiving ADHD pharmacological treatment.
5. *Descriptive information*: type of diagnosis (based on diagnostic questionnaire, rating scale, answering questions similar to “does any doctor has diagnosed you [or your familiar] with ADHD?,” and records from medical files or registers of health care agencies), the presence of comorbidities, Type (stimulant/nonstimulant) and the generic name of medication (Dexamphetamine, Dexmethylphenidate, Lisdexamfetamine, Mixed Salts Amphetamine, Methylphenidate Immediate and Extended Release, Atomoxetine), and level of care (primary, secondary or tertiary).

### 3.5 Assessing of study quality

We will use a modified version of the Newcastle–Ottawa scale (NOS),^[41]^ which is one of the most used methods to evaluate study quality of nonrandomized studies, as described in the Cochrane Handbook.^[42]^ With reference to a modified version used elsewhere,^[43]^ we will use the first 2 domains for the selection of the study groups and the comparability of the groups, as the third domain (exposure) does not fit for systematic reviews of prevalence studies. All four subitens from the selection domain can be rated 0 up to 1 point, whereas the comparability can be rated 0 up to 2 points. In total, each study can receive a minimum of zero (low quality, high risk of bias) to a maximum of six points (high quality, low risk of bias) (Supplemental Digital Content 5)

Two authors will independently access and score individually each study included. In a second step, results will be compared and in case of discordances without agreement, a third author (CRMM) will act as an arbitrator.

### 3.6 Data synthesis

The authors will present a qualitative synthesis of data collected, a PRISMA flowchart, and tables. Additionally, the results of the meta-analysis and meta-regression analysis, if feasible, will be presented in the main text, forest plots and tables, respectively.

To deal with incomplete or missing data, we will contact study authors to ask for additional information. However, to avoid zero cases, the Cochrane Collaboration-recommended approach of including 0.5 will be applied.^[42]^

Meta-analysis of odds ratios (OR) will be performed with the Meta package of R software, and normality tests will be accomplished. If feasible, results will be presented as adjusted and unadjusted ORs. We will first perform normality tests with the study rates, using Log, Logit, and Freeman-Tukey Double Arcsine transformation.^[44]^ Next, according to the distribution rate of normality tests, the best estimation method will be chosen and the Metaprop function will compute the independent prevalence rate and 95% confidence interval. As heterogeneity between the selected studies is expected, we will conduct a meta-analysis with the DerSimonian and Laird method of random-effect model^[45]^ using the pooled prevalence of pharmacological treated and untreated ADHD subjects. Heterogeneity between studies in the meta-analysis will be evaluated with the Cochran's chi-squared test (Cochran's Q), and the *I*^2^ where the interpretation is the following:^[46]^

0%–40%: heterogeneity might not be important
30%–60%: may represent moderate heterogeneity
50%–90%: may represent substantial heterogeneity
75%–100%: substantial heterogeneity.

Publication bias will be explored through the visual inspection of the funnel plot asymmetry, and Egger's linear regression test.^[47]^

#### 3.6.1 Exploring sources of heterogeneity

To explore the effect of individual studies in the prevalence rate and in heterogeneity, we will use the jackknife sensitivity analysis. The jackknife method is a usual procedure in the meta-analysis to test the stability of the results. The procedure is done by computing the results of the meta-analysis removing a different study at a time and then repeating the analyses.^[48]^

In addition, we will conduct a random-effects meta-regression analysis using the pooled prevalence of ADHD pharmacological treatment of ADHD subjects. The covariates to be used will be: continent where the study was performed, age group (children, adolescents, and adults), the method of diagnosis (diagnostic instrument, answering questions similar to “did any doctor diagnose you [or your relative] with ADHD?,” or a clinical diagnosis established by a doctor: GP (general practitioner), pediatrician, or a psychiatrist), diagnostic criteria (any version of DSM or ICD), class of pharmacological treatment (stimulant/nonstimulant), level of care (primary, secondary or tertiary), and study quality (rating at the Newcastle Ottawa Scale). The meta-regression analysis will be conducted with R and STATA 13.0 software.

## 4 Ethics and dissemination

This is a systematic review and meta-analysis; therefore, no IRB (Institutional Review Board) approvals will be necessary. The competing interests are reported in the appropriate item, where all the authors stated their conflict of interests. The results of this study will be published in peer-reviewed journals, and conferences.

## Author contributions

CRMM conceived the study and drafted the protocol. RM, LT, GCAM, FC, SC, and LAR, contributed to the protocol design. CRMM and SC conceived the search strategy. All authors contributed to the development of inclusion and exclusion criteria. All authors read, contributed, and approved the final manuscript.

**Conceptualization:** Carlos Renato Moreira-Maia, Samuele Cortese, Luis Augusto Rohde.

**Investigation:** Rafael Massuti, Luca Tessari, Fausto Campani, Glaucia Chiyoko Akutagava-Martins.

**Methodology:** Carlos Renato Moreira-Maia, Samuele Cortese, Luis Augusto Rohde.

**Project administration:** Carlos Renato Moreira-Maia.

**Writing – original draft:** Carlos Renato Moreira-Maia, Samuele Cortese, Luis Augusto Rohde.

**Writing – review & editing:** Rafael Massuti, Luca Tessari, Fausto Campani, Glaucia Chiyoko Akutagava-Martins.

## Supplementary Material

Supplemental Digital Content
