# Are ADHD medications under or over prescribed worldwide?: Protocol for a systematic review and meta-analysis: Erratum

**DOI:** 10.1097/MD.0000000000011472

**Published:** 2018-07-06

## Abstract

**Background:**

Attention-Deficit/Hyperactivity Disorder (ADHD) is a common neurodevelopmental disorder, characterized by age inappropriate and impairing levels of inattention and/or hyperactivity/impulsivity. Pharmacotherapy is an important part of the ADHD multimodal treatment. The extent to which ADHD is pharmacologically over or under treated worldwide is controversial. We aimed to estimate the pooled worldwide rate of ADHD pharmacological treatment in individuals with and without the disorder.

**Methods:**

We will include published or unpublished studies reporting the rates of ADHD pharmacological treatment in participants with and without ADHD of any age group. Population-based, cohort, or follow-up studies, as well as data from insurance health system and third-party reimbursements will be eligible. Searches will be performed in a large number of electronic databases, including Medline, Embase, CINAHL, Cochrane, PsycINFO, Web of Science, and Scopus. The primary outcome will be the prevalence of ADHD pharmacological treatment in individuals with ADHD and without ADHD. Two independent reviewers will perform the screening, and data extraction process. Study quality/bias will be assessed with the Newcastle–Ottawa scale by 2 independent reviewers. To test the robustness of the findings, we will perform a series of sensitivity and meta-regression analysis. Analyses will be performed with R and STATA software.

**Results, and Conclusion:**

No IRB approval will be necessary. The results of this systematic review and meta-analysis will be presented at international conferences and published in peer-reviewed journals.

In the article, “Are ADHD medications under or over prescribed worldwide?: Protocol for a systematic review and meta-analysis”,^[1]^ which appeared in Volume 97, Issue 24 or *Medicine*, the authors would like the below corrections noted.

Dr. Luca Tessari's degree should just be MD and Dr. Glaucia Chiyoko Akutagava-Martins’ degree should just be PhD.

The abstract should be:

The below funding information should appear as:

This study was supported by Fundo de Incentivo à Pesquisa of Hospital de Clínicas de Porto Alegre (FIPE/HCPA), and Programa de Pós-Graduação em Ciências Médicas: Psiquiatria from Federal University of Rio Grande do Sul (PPG-P/UFRGS).
FC receives scholarship from Conselho Nacional de Desenvolvimento Científico e Tecnológico (CNPq).
RM receives scholarship from Coordenação de Aperfeiçoamento de Pessoal de Nível Superior (CAPES).
LT, and SC have no competing interests.
In section 3.3.1, the second sentence should appear as “Selected titles will have the full text included for a careful reading by 4 independent reviewers (GCAM, FC, LT, and RM).”
In section 3.3.2, the second sentence should appear as “Independent reviewers working in pairs (FC, LT and RM) will collect and compare the data extraction sheets.”

The following references should have appeared as:

[9] Canadian Attention Deficit Hyperactivity Disorder Resource Alliance (CADDRA). Canadian ADHD Practice Guidelines. In: CADDRA, ed. Third Edition ed. Toronto ON2011: CADDRA. Available at: https://caddra.ca/pdfs/caddraGuidelines2011.pdf. Accessed May 5, 2018.
[17] National Collaborating Centre for Mental Health. Attention Deficit Hyperactivity Disorder: Diagnosis and Management of ADHD in Children, Young People and Adults. Leicester (UK): The British Psychological Society 2009.
