# Supplementary material for: Are ADHD medications under or over prescribed worldwide? Protocol for a systematic review and meta-analysis
Source: Medicine (Baltimore). 2018 Jun 15;97(24):e10923. doi: 10.1097/MD.0000000000010923 (PMC6023876; doi:10.1097/MD.0000000000010923)
Supplement: Supplemental Digital Content [file medi-97-e10923-s001.doc]

Supplemental Digital Content 1: Diagnostic instruments and rating scales accepted for inclusion in the systematic review.

| Diagnosis instrument | Reference |
| --- | --- |
| Child and Adolescent Psychiatric Assessment (CAPA) | Angold A, Costello EJ. The Child and Adolescent Psychiatric Assessment (CAPA). Journal of the American Academy of Child and Adolescent Psychiatry 2000;**39**(1):39-48 doi: 10.1097/00004583-200001000-00015. |
| Child Symptom Inventory-4 | Gadow KD, Sprafkin JN. *Child symptom inventory 4: Screening and norms manual*: Checkmate Plus, 2002. |
| Composite international diagnostic interview (CIDI) | Robins LN, Wing J, Wittchen HU, et al. The Composite International Diagnostic Interview. An epidemiologic Instrument suitable for use in conjunction with different diagnostic systems and in different cultures. Archives of general psychiatry 1988;**45**(12):1069-77. |
| Development and Well-being Assessment (DAWBA) | Goodman R, Ford T, Richards H, et al. The Development and Well-Being Assessment: description and initial validation of an integrated assessment of child and adolescent psychopathology. Journal of child psychology and psychiatry, and allied disciplines 2000;**41**(5):645-55. |
| Diagnostic Interview Schedule for Children (DISC) | Shaffer D, Fisher P, Lucas CP, et al. NIMH Diagnostic Interview Schedule for Children Version IV (NIMH DISC-IV): description, differences from previous versions, and reliability of some common diagnoses. Journal of the American Academy of Child and Adolescent Psychiatry 2000;**39**(1):28-38 doi: 10.1097/00004583-200001000-00014. |
| Schedule for Affective Disorders and Schizophrenia for School-age children ADHD module (K-SADS) | Kaufman J, Birmaher B, Brent D, et al. Schedule for Affective Disorders and Schizophrenia for School-Age Children-Present and Lifetime Version (K-SADS-PL): initial reliability and validity data. Journal of the American Academy of Child and Adolescent Psychiatry 1997;**36**(7):980-8 doi: 10.1097/00004583-199707000-00021. |
| Mini-International Neuropsychiatric Interview-Plus (MINI-Plus)[7] | Sheehan DV, Lecrubier Y, Sheehan KH, et al. The Mini-International Neuropsychiatric Interview (M.I.N.I.): the development and validation of a structured diagnostic psychiatric interview for DSM-IV and ICD-10. The Journal of clinical psychiatry 1998;**59 Suppl 20**:22-33;quiz 34-57. |
| Amsterdam Diagnostisch Interview voor Kinderen (ADIKA) | Kortenbout Van der Sluijs M, Levita Dd, Manen Rv, et al. Amsterdam Diagnostisch Interview voor Kinderen en Adolescenten (ADIKA)[Amsterdam Diagnostic Interview for Children and Adolescents]. Lisse: Swets en Zeitlinger 1993. |
| Diagnostic Interview for Children and Adolescents (DICA) | Welner Z, Reich W, Herjanic B, et al. Reliability, validity, and parent-child agreement studies of the Diagnostic Interview for Children and Adolescents (DICA). Journal of the American Academy of Child and Adolescent Psychiatry 1987;**26**(5):649-53 doi: 10.1097/00004583-198709000-00007. |
| Ontario Child Health Study (OCHS) Hyperactivity Scale | Boyle MH, Offord DR, Racine Y, et al. Evaluation of the revised Ontario Child Health Study scales. Journal of child psychology and psychiatry, and allied disciplines 1993;**34**(2):189-213 |
| Structured Clinical Interview for DSM-III-R (SCID) | Williams JB, Gibbon M, First MB, et al. The Structured Clinical Interview for DSM-III-R (SCID). II. Multisite test-retest reliability. Archives of general psychiatry 1992;**49**(8):630-6. |
| Strengths and difficulties questionnaire (SDQ) | Stone LL, Otten R, Engels RC, et al. Psychometric properties of the parent and teacher versions of the strengths and difficulties questionnaire for 4- to 12-year-olds: a review. Clinical child and family psychology review 2010;**13**(3):254-74 doi: 10.1007/s10567-010-0071-2. |

| Supplemental Digital Content 2: Diagnostic instruments and rating scales accepted for inclusion in the systematic review | |
| --- | --- |
| Rating scales | Reference |
| ADHD Rating Scale (DuPaul) | DuPaul G, Power T, Anastopoulos A, et al. *ADHD rating scale II: checklists, norms, and clinical interpretation*. Guilford, New York, 1998. |
| Adult ADHD Investigator System Report Scale (AISRS) | Spencer TJ, Adler LA, Meihua Q, et al. Validation of the adult ADHD investigator symptom rating scale (AISRS). Journal of attention disorders 2010;**14**(1):57-68 doi: 10.1177/1087054709347435. |
| Adult ADHD Self-Report Scale (ASRS-v1.1) | Adler L, Kessler R, Spencer T. Adult ADHD Self-Report Scale-v 1.1 (ASRS-v1. 1) Symptom Checklist. New York, NY, 2004. |
| Adult ADHD Self-Report Scale-Screening (ASRS-S) | Kessler RC, Adler L, Ames M, et al. The World Health Organization Adult ADHD Self-Report Scale (ASRS): a short screening scale for use in the general population. Psychological medicine 2005;**35**(2):245-56. |
| Brief Psychiatric Rating Scale for Children (BPRS-C) | Gale J, Pfefferbaum B, Suhr M, et al. The Brief Psychiatric Rating Scale for Children: A Reliability Study. Journal of Clinical Child Psychology 1986;**15**(4):341 – 45. |
| Conners, Loney, and Milich Scale (CLAM) | Conners CK. *Conners' rating scales revised*: Multi-Health Systems, Incorporated, 2001. |
| Conners' ADHD/DSM IV scales (CADS) | Conners CK. *Conners' rating scales revised*: Multi-Health Systems, Incorporated, 2001. |
| Conners' parent rating scale (CPRS-R) | Conners CK. *Conners' rating scales revised*: Multi-Health Systems, Incorporated, 2001. |
| Conners' teacher rating scale (CTRS-R) | Conners CK. *Conners' rating scales revised*: Multi-Health Systems, Incorporated, 2001. |
| Conners' Abbreviated rating scale (ABRS) | Conners CK. *Conners' rating scales revised*: Multi-Health Systems, Incorporated, 2001. |
| Conners' Abbreviated symptom questionnaire (ASQ) | Conners CK. *Conners' rating scales revised*: Multi-Health Systems, Incorporated, 2001. |
| Conners' Global Index for Parents (CGI parents) | Conners CK. *Conners' rating scales revised*: Multi-Health Systems, Incorporated, 2001. |
| Conners' Global Index for Teachers (CGI teacher) | Conners CK. *Conners' rating scales revised*: Multi-Health Systems, Incorporated, 2001. |
| Conners' hyperkinetic index | Conners CK. *Conners' rating scales revised*: Multi-Health Systems, Incorporated, 2001. |
| Conners'-Wells Adolescent Self-Report of Symptoms Scale | Conners CK. *Conners' rating scales revised*: Multi-Health Systems, Incorporated, 2001. |
| Conners Adult ADHD Rating Scales (CAARS) | Conners CK. *Conners' rating scales revised*: Multi-Health Systems, Incorporated, 2001. |
| Fremdbeurteilungsbogen für Hyperkinetische Störungen (FBB HKS) | Brühl B, Döpfner M, Lehmkuhl G. Der Fremdbeurteilungsbogen für hyperkinetische Störungen (FBB-HKS)–Prävalenz hyperkinetischer Störungen im Elternurteil und psychometrische Kriterien. Kindheit und Entwicklung 2000;**9**(2):115-25 |
| Conners rating scale (parent) (IOWA) | Loney J, Milich R. Hyperactivity, inattention, and aggression in clinical practice. Advances in developmental and behavioral pediatrics 1982;**3**(1):113-47 |
| Conners rating scale (teacher) (IOWA) | Pelham Jr WE, Milich R, Murphy DA, et al. Normative data on the IOWA Conners teacher rating scale. Journal of Clinical Child Psychology 1989;**18**(3):259-62. |
| Parental Account of Children’s Symptoms (PACS) | Taylor E, Schachar R, Thorley G, et al. Conduct disorder and hyperactivity: I. Separation of hyperactivity and antisocial conduct in British child psychiatric patients. The British journal of psychiatry : the journal of mental science 1986;**149**:760-7 |
| Swanson, Nolan, and Pelham-IV Questionnaire (SNAP-IV) | Swanson JM, Kraemer HC, Hinshaw SP, et al. Clinical relevance of the primary findings of the MTA: success rates based on severity of ADHD and ODD symptoms at the end of treatment. Journal of the American Academy of Child and Adolescent Psychiatry 2001;**40**(2):168-79 doi: 10.1097/00004583-200102000-00011. |
| Swanson, Kotkin, Atkins, MFlynn, Pelham Scale (SKAMP) | Wigal SB, Gupta S, Guinta D, et al. Reliability and validity of the SKAMP rating scale in a laboratory school setting. Psychopharmacology bulletin 1998;**34**(1):47. |
| Strengths and Weaknesses of ADHD Symptoms and Normal Behaviors (SWAN) | Swanson JM, Schuck S, Porter MM, et al. Categorical and Dimensional Definitions and Evaluations of Symptoms of ADHD: History of the SNAP and the SWAN Rating Scales. The International journal of educational and psychological assessment 2012;**10**(1):51-70. |
| Teacher Self Control Rating Scale (TSCRS) | Kendall PC, Zupan BA, Braswell L. Self-control in children: Further analyses of the Self-Control Rating Scale. Behavior Therapy 1981;**12**(5):667-81. |
| The ADD/H Comprehensive Teacher Rating Scale (ACTeRS) | Ullmann RK, Sleator EK, Sprague R. The ADD/H Comprehensive Teacher Rating Scale (ACTeRS). Odessa, FL: Psychological Assessment Resources 1991. |
| Vanderbilt ADHD Teacher Rating Scale (VARTRS) | Wolraich ML, Lambert W, Doffing MA, et al. Psychometric properties of the Vanderbilt ADHD diagnostic parent rating scale in a referred population. Journal of pediatric psychology 2003;**28**(8):559-68 |
| Vanderbilt ADHD Diagnostic Parent Rating Scale (VADPRS) | Wolraich ML, Lambert W, Doffing MA, et al. Psychometric properties of the Vanderbilt ADHD diagnostic parent rating scale in a referred population. Journal of pediatric psychology 2003;**28**(8):559-68 |
| Wender-Reimherr Adult Attention Deficit Disorder Scale (WRAADDS) | Rosler M, Retz W, Retz-Junginger P, et al. Attention deficit hyperactivity disorder in adults. Benchmarking diagnosis using the Wender-Reimherr adult rating scale. Der Nervenarzt 2008;**79**(3):320-7 doi: 10.1007/s00115-007-2375-0. |
| Abbreviations  ADD/H: Attention-Deficit Disorder with Hyperactivity; ADHD: Attention-Deficit/Hyperactivity Disorder; | |

**Supplemental Digital Content 3: Databases Search strategies for the systematic review**

**Medline 1964 to present**

1 "minimal brain disorder" OR "minimal brain dysfunction" (530)

2 "overactive n3 child n3 syndrome" (0)

3 adhd OR ADHD OR addh OR ADD (67,107)

4 attention n3 deficit n3 (disorder* OR syndrome*) n6 (hyperactiv* OR hyperkinetic*) (28,019)

5 (hyperkinetic* OR hyperactivit*) n3 (disorder OR syndrome) (28,599)

6 (MH "Attention Deficit Disorder with Hyperactivity) (22,334)

7 S1 OR S2 OR S3 OR S4 OR S5 OR S6 (79,407)

8 Amphetamine* OR Amfetamine* OR Dextroamphetamine* OR Dexamphetamine* OR "Mixed amphetamine salts" OR Lisdexamfetamine* OR Methylphenidate OR Atomoxetine OR Clonidine OR guanfacine OR stimulant* OR psychostimulant* OR Elvanse OR Venvanse OR Adderall OR Dexedrine OR Detrostat OR Vyvanse OR ProCentra OR Dyanavel OR Evekeo OR Zenzedi OR Desoxyn OR Metadate OR Concerta OR Daytrana OR Ritalin OR Methylin OR Quillivant OR Focalin OR Biphentin OR Phenida OR Ritalina OR Hynidate OR Addwize OR Inspiral OR Attenade OR Medikinet OR Equasym OR Penid OR Tranquilyn OR Rubifen OR Aptensio OR Strattera OR Tomoxetin OR Attentrol OR Axepta OR Atoken OR Attentin OR Kapvay OR Intuniv (85,840)

9 "pharmacological treatment*" OR drug* n3 treatment* OR pharmacotherapy OR psychotropic n3 drug* OR medicat* OR (MH “Psychotropic Drugs”) (389,390)

10 S8 OR S9 (465,070)

11 (cohort n (study or studies)) OR "cohort analy*" OR (follow up n (study or studies)) OR (observational n (study or studies)) OR longitudinal OR retrospective OR (epidemiological n (study OR studies)) OR ("cross section" n (study OR studies)) OR "cross sectional" OR "follow up" OR (MH Health Care Surveys) (2,210,744)

12 incidence OR prevalence OR occur* OR frequenc* OR proportion* OR rate* OR number* OR percent* OR episode* OR epidemiolo* OR distribut* OR demograph* OR survey* OR trend* (8,438,607)

13 (MH "Epidemiologic Methods+") (4,871,843)

14 (MH "Incidence") (202,227)

15 (MH "Prevalence") (216,475)

16 (MH "Demography") (54,925)

17 (MH Epidemiology+) (22,841)

18 S11 OR S12 OR S13 OR S14 OR S15 OR S16 OR S17 (10,514,770)

19 S7 AND S10 AND S18 (8,430)

20 S7 and S10 (12,234)

**CINAHL 1937 to present**

1 "minimal brain disorder" OR "minimal brain dysfunction" (14)

2 "overactive n3 child n3 syndrome" (0)

3 adhd OR ADHD OR addh OR ADD (19,038)

4 attention n3 deficit n3 (disorder* OR syndrome*) n6 (hyperactiv* OR hyperkinetic*) (12,048)

5 (hyperkinetic* OR hyperactivit*) n3 (disorder OR syndrome) (12,176)

6 (MH "Attention Deficit Hyperactivity Disorder") (10,925)

7 S1 OR S2 OR S3 OR S4 OR S5 OR S6 (25,344)

8 Amphetamine* OR Amfetamine* OR Dextroamphetamine* OR Dexamphetamine* OR "Mixed amphetamine salts" OR Lisdexamfetamine* OR Methylphenidate OR Atomoxetine OR Clonidine OR guanfacine OR stimulant* OR psychostimulant* OR Elvanse OR Venvanse OR Adderall OR Dexedrine OR Detrostat OR Vyvanse OR ProCentra OR Dyanavel OR Evekeo OR Zenzedi OR Desoxyn OR Metadate OR Concerta OR Daytrana OR Ritalin OR Methylin OR Quillivant OR Focalin OR Biphentin OR Phenida OR Ritalina OR Hynidate OR Addwize OR Inspiral OR Attenade OR Medikinet OR Equasym OR Penid OR Tranquilyn OR Rubifen OR Aptensio OR Strattera OR Tomoxetin OR Attentrol OR Axepta OR Atoken OR Attentin OR Kapvay OR Intuniv (9,105)

9 "pharmacological treatment*" OR drug* n3 treatment* OR pharmacotherapy OR psychotropic n3 drug* OR medicat* (99.895)

10 (MH "Psychotropic Drugs" ) ( 4,229)

11 S8 OR S9 OR S10 (106,878)

12 (cohort n (study or studies)) OR "cohort analy*" OR (follow up n (study or studies)) OR (observational n (study or studies)) OR longitudinal OR retrospective OR (epidemiological n (study OR studies)) OR ("cross section" n (study OR studies)) OR "cross sectional" OR "follow up" (418,102)

13 incidence OR prevalence OR occur* OR frequenc* OR proportion* OR rate* OR number* OR percent* OR episode* OR epidemiolo* OR distribut* OR demograph* OR survey* OR trend* (1,813,918)

14 (MH "Epidemiology+") (449,399)

15 (MH "Incidence") (41,327)

16 (MH "Prevalence") (56, 241)

17 (MH "Demography") (28, 939)

18 S12 OR S13 OR S14 OR S15 OR S16 OR S17 (1,469,900)

19 S7 AND S11 AND S18 (2,171)

**PsychInfo 1656 to present**

1 minimal brain disorder" OR "minimal brain dysfunction" (14)

2 "overactive n3 child n3 syndrome" (0)

3 adhd OR ADHD OR addh OR ADD (51,211)

4 attention n3 deficit n3 (disorder* OR syndrome*) n6 (hyperactiv* OR hyperkinetic*) (24,282)

5 (hyperkinetic* OR hyperactivit*) n3 (disorder OR syndrome) (25,282)

6 DE "Attention Deficit Disorde”r OR DE “Attention Deficit Disorder with Hyperactivity” (21,559)

7 S1 OR S2 OR S3 OR S4 OR S5 OR S6 (57,732)

8 Amphetamine* OR Amfetamine* OR Dextroamphetamine* OR Dexamphetamine* OR "Mixed amphetamine salts" OR Lisdexamfetamine* OR Methylphenidate OR Atomoxetine OR Clonidine OR guanfacine OR stimulant* OR psychostimulant* OR Elvanse OR Venvanse OR Adderall OR Dexedrine OR Detrostat OR Vyvanse OR ProCentra OR Dyanavel OR Evekeo OR Zenzedi OR Desoxyn OR Metadate OR Concerta OR Daytrana OR Ritalin OR Methylin OR Quillivant OR Focalin OR Biphentin OR Phenida OR Ritalina OR Hynidate OR Addwize OR Inspiral OR Attenade OR Medikinet OR Equasym OR Penid OR Tranquilyn OR Rubifen OR Aptensio OR Strattera OR Tomoxetin OR Attentrol OR Axepta OR Atoken OR Attentin OR Kapvay OR Intuniv (26,951)

9 "pharmacological treatment*" OR drug* n3 treatment* OR pharmacotherapy OR psychotropic n3 drug* OR medicat* (109,979)

10 S8 OR S9 (130,898)

11 (cohort n (study or studies)) OR "cohort analy*" OR (follow up n (study or studies)) OR (observational n (study or studies)) OR longitudinal OR retrospective OR (epidemiological n (study OR studies)) OR ("cross section" n (study OR studies)) OR "cross sectional" OR "follow up" (247,494)

12 incidence OR prevalence OR occur* OR frequenc* OR proportion* OR rate* OR number* OR percent* OR episode* OR epidemiolo* OR distribut* OR demograph* OR survey* OR trend* (1,383,556)

13 DE "Epidemiology) (42,989)

14 S11 OR S12 OR S13 (1,490,917)

15 S7 AND S10 AND S14 (4,437)

**Embase 1996 to present**

1 (("minimal brain disorder" or "minimal brain dysfunction" or "overactive ADJ3 child ADJ3 syndrome" or (attention adj3 deficit adj3 (disorder* or syndrome*) adj6 (hyperactiv* or hyperkinetic*))) and ((hyperkinetic* or hyperactivit*) adj3 (disorder or syndrome))).af. (22903)

2 attention deficit disorder/ (40958)

3 1 or 2 (43967)

4 ((cohort adj1 (study or studies)) or "cohort analy*" or (follow up adj1 (study or studies)) or (observational adj1 (study or studies)) or longitudinal or retrospective or (epidemiological adj1 (study or studies)) or ("cross section" adj1 (study or studies)) or "cross sectional" or "follow up" or (incidence or prevalence or occur* or frequenc* or proportion* or rate* or number* or percent* or episode* or epidemiolo* or distribut* or demograph* or survey* or trend*)).af. (8557866)

5 health care survey/ (10379)

6 incidence/ (227152)

7 prevalence/ (451729)

8 demography/ (129483)

9 epidemiology/ (88820)

10 5 or 6 or 7 or 8 or 9 (839708)

11 4 or 10 (8557866)

12 (Amphetamine* or Amfetamine* or Dextroamphetamine* or Dexamphetamine* or "Mixed amphetamine salts" or Lisdexamfetamine* or Methylphenidate or Atomoxetine or Clonidine or guanfacine or stimulant* or psychostimulant* or Elvanse or Venvanse or Adderall or Dexedrine or Detrostat or Vyvanse or ProCentra or Dyanavel or Evekeo or Zenzedi or Desoxyn or Metadate or Concerta or Daytrana or Ritalin or Methylin or Quillivant or Focalin or Biphentin or Phenida or Ritalina or Hynidate or Addwize or Inspiral or Attenade or Medikinet or Equasym or Penid or Tranquilyn or Rubifen or Aptensio or Strattera or Tomoxetin or Attentrol or Axepta or Atoken or Attentin or Kapvay or Intuniv or ((((("pharmacological treatment*" or drug*) adj3 treatment*) or pharmacotherapy or psychotropic) adj3 drug*) or medicat*)).af. (798043)

13 psychotropic agent/ (18522)

14 12 or 13 (806092)

15 3 and 11 and 14 (8507)

**Web of Science 1970 to present**

You searched for: **TOPIC: ("minimal brain disorder" OR "minimal brain dysfunction" OR "overactive n3 child n3 syndrome" ORadhd OR ADHD OR addh OR ADD ORattention n3 deficit n3 (disorder* OR syndrome*) n6 (hyperactiv* OR hyperkinetic*) OR (hyperkinetic* OR hyperactivit*) n3 (disorder OR syndrome)) *AND* TOPIC: ("pharmacological treatment*" OR drug* n3 treatment* OR pharmacotherapy OR psychotropic n3 drug* OR medicat* OR Amphetamine* OR Amfetamine* OR Dextroamphetamine* OR Dexamphetamine* OR "Mixed amphetamine salts" OR Lisdexamfetamine* OR Methylphenidate OR Atomoxetine OR Clonidine OR guanfacine OR stimulant* OR psychostimulant* OR Elvanse OR Venvanse OR Adderall OR Dexedrine OR Detrostat OR Vyvanse OR ProCentra OR Dyanavel OR Evekeo OR Zenzedi OR Desoxyn OR Metadate OR Concerta OR Daytrana OR Ritalin OR Methylin OR Quillivant OR Focalin OR Biphentin OR Phenida OR Ritalina OR Hynidate OR Addwize OR Inspiral OR Attenade OR Medikinet OR Equasym OR Penid OR Tranquilyn OR Rubifen OR Aptensio OR Strattera OR Tomoxetin OR Attentrol OR Axepta OR Atoken OR Attentin OR Kapvay OR Intuniv) *AND* TOPIC: ((cohort near1 (study or studies)) OR "cohort analy*" OR (follow up near1 (study or studies)) OR (observational near1 (study or studies)) OR longitudinal OR retrospective OR (epidemiological near1 (study OR studies)) OR ("cross section" near1 (study OR studies)) OR "cross sectional" OR "follow up" OR incidence OR prevalence OR occur* OR frequenc* OR proportion* OR rate* OR number* OR percent* OR episode* OR epidemiolo* OR distribut* OR demograph* OR survey* OR trend*)** [**...More**](javascript:void(0))

TOPIC: ("minimal brain disorder" OR "minimal brain dysfunction" OR "overactive near3 child near3 syndrome" OR adhd OR ADHD OR addh OR ADD OR attention near3 deficit near3 (disorder* OR syndrome*) near6 (hyperactiv* OR hyperkinetic*) OR (hyperkinetic* OR hyperactivit*) near3 (disorder OR syndrome))

*AND*

TOPIC: "pharmacological treatment*" OR drug* near3 treatment* OR pharmacotherapy OR psychotropic near3 drug* OR medicat* OR Amphetamine* OR Amfetamine* OR Dextroamphetamine* OR Dexamphetamine* OR "Mixed amphetamine salts" OR Lisdexamfetamine* OR Methylphenidate OR Atomoxetine OR Clonidine OR guanfacine OR stimulant* OR psychostimulant* OR Elvanse OR Venvanse OR Adderall OR Dexedrine OR Detrostat OR Vyvanse OR ProCentra OR Dyanavel OR Evekeo OR Zenzedi OR Desoxyn OR Metadate OR Concerta OR Daytrana OR Ritalin OR Methylin OR Quillivant OR Focalin OR Biphentin OR Phenida OR Ritalina OR Hynidate OR Addwize OR Inspiral OR Attenade OR Medikinet OR Equasym OR Penid OR Tranquilyn OR Rubifen OR Aptensio OR Strattera OR Tomoxetin OR Attentrol OR Axepta OR Atoken OR Attentin OR Kapvay OR Intuniv

*AND*

TOPIC: ((cohort near1 (study or studies)) OR "cohort analy*" OR (follow up near1 (study or studies)) OR (observational near1 (study or studies)) OR longitudinal OR retrospective OR (epidemiological near1 (study OR studies)) OR ("cross section" near1 (study OR studies)) OR "cross sectional" OR "follow up" OR incidence OR prevalence OR occur* OR frequenc* OR proportion* OR rate* OR number* OR percent* OR episode* OR epidemiolo* OR distribut* OR demograph* OR survey* OR trend*

**Scopus 1996 to present**

Article Title, Abstract, Keywords ("minimal brain disorder" OR "minimal brain dysfunction" OR "overactive W/3 child W/3 syndrome" OR adhd OR ADHD OR addh OR ADD OR attention W/3 deficit W/3 (disorder* OR syndrome*) W/6 (hyperactiv* OR hyperkinetic*) OR (hyperkinetic* OR hyperactivit*) W/3 (disorder OR syndrome))

*AND*

Article Title, Abstract, Keywords**:** "pharmacological treatment*" OR drug* W/3 treatment* OR pharmacotherapy OR psychotropic W/3 drug* OR medicat* OR Amphetamine* OR Amfetamine* OR Dextroamphetamine* OR Dexamphetamine* OR "Mixed amphetamine salts" OR Lisdexamfetamine* OR Methylphenidate OR Atomoxetine OR Clonidine OR guanfacine OR stimulant* OR psychostimulant* OR Elvanse OR Venvanse OR Adderall OR Dexedrine OR Detrostat OR Vyvanse OR ProCentra OR Dyanavel OR Evekeo OR Zenzedi OR Desoxyn OR Metadate OR Concerta OR Daytrana OR Ritalin OR Methylin OR Quillivant OR Focalin OR Biphentin OR Phenida OR Ritalina OR Hynidate OR Addwize OR Inspiral OR Attenade OR Medikinet OR Equasym OR Penid OR Tranquilyn OR Rubifen OR Aptensio OR Strattera OR Tomoxetin OR Attentrol OR Axepta OR Atoken OR Attentin OR Kapvay OR Intuniv

*AND*

Article Title, Abstract, Keywords**:** ((cohort W/1 (study or studies)) OR "cohort analy*" OR (follow up W/1 (study or studies)) OR (observational W/1 (study or studies)) OR longitudinal OR retrospective OR (epidemiological W/1 (study OR studies)) OR ("cross section" W/1 (study OR studies)) OR "cross sectional" OR "follow up" OR incidence OR prevalence OR occur* OR frequenc* OR proportion* OR rate* OR number* OR percent* OR episode* OR epidemiolo* OR distribut* OR demograph* OR survey* OR trend*

**Supplemental Digital Content 4: List of websites**

Prospero

Cochrane Library

Campbell Collection of Systematic reviews

FDA U.S. Food and Drug Administration

Dept. of Health

Proquest: Dissertations and Thesis: UK and Ireland.

Google (using time limits of 24 hours/past week) to pick up in press articles

Evidence search

CRD – Centers for Reviews and Dissemination

NICE

Medlar

Open Grey

Grey literature in Public Health

Oaister

Zetoc

NTIS – National Technical Information Service

Trip – Turning Research into Practice

Grey literature Report

NICHSR (Natl Info Center on Health Services Research and Health Care Technology)

RePORT (Research Portfolio Online Reporting Tools)

CADTH checklist

ETHOS (Electronic Theses Online Service)

DART – Europe E-Theses portal

WHO

Public Health England

**Supplemental Digital Content 5: Assessment of study quality and risk of bias**

|  | **Newcastle-Ottawa Scale (NOS) - CASE CONTROL** | | | | | |
| --- | --- | --- | --- | --- | --- | --- |
|  | Selection (OK = 1 point) | | | | Comparability  (Up to 2 points) |  |
|  | 1) Definition | 2) Representativeness | 3) Controls | 4) Definition of Controls |  | Total |
| Reviewer 1 |  |  |  |  |  |  |
| Reviewer 2 |  |  |  |  |  |  |

1) Is the case definition adequate?

a) yes, with independent validation (1 point) -  >1 person/record/time/process to extract information, or reference to primary record source such as x-rays or medical/hospital records

b) yes, e.g. record linkage or based on self-reports - e.g. ICD (International Classification of Diseases) codes in database or self-report with no reference to primary record or no description

c) no description

2) Representativeness of the cases

a) consecutive or obviously representative series of cases (1 point)

b) potential for selection biases or not stated

3) Selection of Controls

a) community controls (1 point)

b) hospital controls

c) no description

4) Definition of Controls

a) no history of disease (endpoint) (1 point)

b) no description of source

5) Comparability of cases and controls on the basis of the design or analysis

a) study controls for ___ (select the most important factor) (1 point)

b) study controls for any additional factor (This criterion could be modified to indicate specific control for a second important factor.) (1 point)

|  | **Newcastle-Ottawa Scale (NOS) - COHORT** | | | | | |
| --- | --- | --- | --- | --- | --- | --- |
|  | Selection (OK = 1 point) | | | | Comparability  (Up to 2 points) |  |
|  | 1) Representativeness | 2) Selection Non-exposed | 3) Ascertainment of Exposure | 4) Outcome not present |  | Total |
| Reviewer 1 |  |  |  |  |  |  |
| Reviewer 2 |  |  |  |  |  |  |

1) Representativeness of the exposed cohort

a) truly representative of the average _____ (describe) in the community (1 point)

b) somewhat representative of the average _____ in the community (1 point)

c) selected group of users e.g. nurses, volunteers

d) no description of the derivation of the cohort

2) Selection of the non-exposed cohort

a) drawn from the same community as the exposed cohort (1 point)

b) drawn from a different source

c) no description of the derivation of the non-exposed cohort

3) Ascertainment of exposure to (e.g.) implants

a) secure record (e.g. surgical records) (1 point)

b) structured interview (1 point)

c) written self-report

d) no description

4) Demonstration that outcome of interest was not present at start of study

a) yes (1 point) - In the case of mortality studies, outcome of interest is still the presence of a disease/ incident, rather than death; that is a statement of no history of disease or incident earns a star

b) no

5) Comparability of cohorts on the basis of the design or analysis

a) study controls for ___ (select the most important factor) (1 point)

b) study controls for any additional factor (This criterion could be modified to indicate specific control for a second important factor.) (1 point)
